# Situational analysis to inform development of primary care and community-based mental health services for severe mental disorders in Nepal

**DOI:** 10.1186/s13033-017-0176-9

**Published:** 2017-11-15

**Authors:** Mangesh Angdembe, Brandon A. Kohrt, Mark Jordans, Damodar Rimal, Nagendra P. Luitel

**Affiliations:** 1 Research Department, Transcultural Psychosocial Organisation (TPO) Nepal, Kathmandu, Nepal; 2 Department of Psychiatry, George Washington University, Washington DC, USA; 3 War Child Holland The Netherlands and Centre for Global Mental Health, Institute of Psychiatry, Psychology and Neuroscience, King’s College London, London, UK

**Keywords:** Community mental health, Severe mental disorders, Task shifting, Stigma, Patient support groups, mHealth

## Abstract

**Background:**

Nepal is representative of Low and Middle Income Countries (LMIC) with limited availability of mental health services in rural areas, in which the majority of the population resides.

**Methods:**

This formative qualitative study explores resources, challenges, and potential barriers to the development and implementation of evidence-based Comprehensive Community-based Mental Health Services (CCMHS) in accordance with the mental health Gap Action Programme (mhGAP) for persons with severe mental health disorders and epilepsy. Focus Group Discussions (FGDs, n = 9) and Key-Informant Interviews (KIIs, n = 26) were conducted in a rural district in western Nepal. Qualitative data were coded using the Framework Analysis Method employing QSR NVIVO software.

**Results:**

Health workers, general community members, and persons living with mental illness typically attributed mental illness to witchcraft, curses, and punishment for sinful acts. Persons with mental illness are often physically bound or locked in structures near their homes. Mental health services in medical settings are not available. Traditional healers are often the first treatment of choice. Primary care workers are limited both by lack of knowledge about mental illness and the inability to prescribe psychotropic medication. Health workers supported upgrading their existing knowledge and skills through mhGAP resources. Health workers lacked familiarity with basic computing and mobile technology, but they supported the introduction of mobile technology for delivering effective mental health services. Persons with mental illness and their family members supported the development of patient support groups for collective organization and advocacy. Stakeholders also supported development of focal community resource persons to aid in mental health service delivery and education.

**Conclusion:**

Health workers, persons living with mental illness and their families, and other stakeholders identified current gaps and barriers related to mental health services. However, respondents were generally supportive in developing community-based care in rural Nepal.

## Background

Mental disorders are frequently occurring and often seriously impairing in many countries across the world [1]. The burden of mental disorders has significantly increased by 37·6% at the duration of 20 years between 1990 and 2010 accounting for 7.4% of disability adjustment life years (DALY), and 22.9% of all years lived with disability [2]. Mental illness is one of the major contributors to global economic burden of non-communicable diseases; it is estimated that the global burden will rise twofold between 2010 and 2030 [3], in part due to the demographic and epidemiological transitions in LMICs [4]. A review study of 174 surveys across 26 high income countries and 37 Low and Middle Income Countries (LMICs) showed that one in five adults (17.6%) experienced a common mental health disorder on average within the past 12 months with a 29.2% chance of experiencing a mental health disorder at a point in their lifetime [5].

According to World Health Organization (WHO), more than 13% of the global burden of disease is due to neuropsychiatric disorders and almost three quarters of this burden lies in low-income and middle-income countries [LMICs] [6]. Nepal is an LMIC that has suffered through a 10-year long conflict (Maoist insurgency), which further increased the mental health burden. Since most LMICs do not routinely conduct their own population-based surveys, several studies on mental health in the general population during and after the Maoist insurgency in Nepal have shown high prevalence rates of mental illness [7–10]. A recent study revealed a 22.7% prevalence of anxiety and 11.7% prevalence of depression in Nepal’s population [11].

The impact of untreated mental disorders results in homelessness, incarceration and violence. It does not only affect the person suffering from the illness but also those close to the person. Studies have shown several adverse consequences of untreated mental illness including poverty [12], physical health problems [13] and premature death [14]. Mental disorders have a negative impact on education and work productivity when compared to persons without mental disorders [15, 16].

More than 40% of all countries have no mental health policies and over 30% have no specific mental health programmes [17]. Several studies have estimated that nearly half of all individuals with severe mental disorders are not receiving treatment for their mental illness at any given time [18–20]. In LMICs, only a minor proportion of people with mental disorders receive adequate treatment [21–25]. Studies show that nearly 80% of people with mental disorders in developing countries receive no effective treatment [6, 21] In many LMICs, there is often only one psychiatrist or psychologist for over two million people [26].

The Government of Nepal has attempted to include mental health services as a basic primary health care component however; services still remain inaccessible to most of the population at the primary care level. The National Health Sector Programme (NHSP-II) of Nepal has included mental health as a part of “essential health care”. However, government policy does not permit primary health care workers to independently diagnose and treat mental disorders within the primary care system. Procedures for referring persons from primary care to secondary/tertiary care also do not exist in the health care system [27].

Stigma is strongly embedded in Nepali communities thereby posing a large challenge in the scaling-up of mental health services. Lack of awareness of mental health services and related issues among both the marginalized and the privileged is another major hindrance. Families and close relatives/friends are primary detectors and identifiers of mental health problems, and can take a pivotal role in making mental health services accessible [28]. The already overburdened health workers, unavailability of psychotropic drugs, lack of awareness in the general people, deeply engrained negative attitudes/stigmatized and discriminating behavior towards the mentally ill all pose as challenges in integrating mental health services in primary health care in Nepal [28–30].

In order to close the gap between mental health needs and services, activists and researchers have initiated a task-sharing (also referred to as ‘task-shifting’) approach, from specialist mental health professionals to non-specialist health workers in LMIC primary health care settings [31–33]. However, the data regarding fidelity of care in task-shifting to community-based services and utilization of mental health services after community training is limited to a few LMIC settings [34].

Integration of mental health services into primary health care is one of most essential health care recommendations from WHO [35]. The integrated approach helps to reduce stigma, improve access to mental health services and treatment of co-morbid physical conditions, reduce chronicity and improve social integration, human rights protection, better health outcomes for people treated in primary health care, and improve human resource capacity for mental health [36].

There have been significant efforts made towards the development of a community mental health delivery integrated program in Nepal [27]. However, no unified model has been adopted or scaled up. This gap highlights the lack of available single best-practice models, as well as the urgent need to better understand how a comprehensive mental health approach, encompassing services within PHC and the surrounding community, can be developed and implemented [28].

## Methods

### Study design

Cross sectional studies involving qualitative methods of data collection and analysis were conducted between December 2012 and May 2013 to portray the prevalent condition of mental disorders, as perceived by district and national levels stakeholders.

### Setting

This formative research was conducted through a program titled Mental Health Beyond Facilities (mhBeF), funded through a grant awarded to Makerere University School of Public Health from Grand Challenges Canada. The objective of mhBeF is to develop and implement an evidence-based comprehensive community-based mental health services (CCMHS) package in accordance with the mental health Gap Action Programme (mhGAP) for persons with severe mental disorders and epilepsy (PWSMDE) in Liberia, Uganda, and Nepal. The CCMHS package integrates three components: (a) strengthening clinical recognition, referral, assessment and management by health workers and community resource persons (CoRPs) including an electronic mobile health (mHealth) package; (b) establishing psychosocial and socio-economic support services for PWSMD through patient support groups (PSGs); and (c) conducting stigma reduction targeted activities for health providers, families and PWSMDE.

As post-conflict countries, Liberia, Uganda, and Nepal each have a high burden of mental disorders and a lack of community-based mental health services. The mhBeF project is implemented in Nepal by Transcultural Psychosocial Organization Nepal (TPO Nepal).

**Fig. 1:**
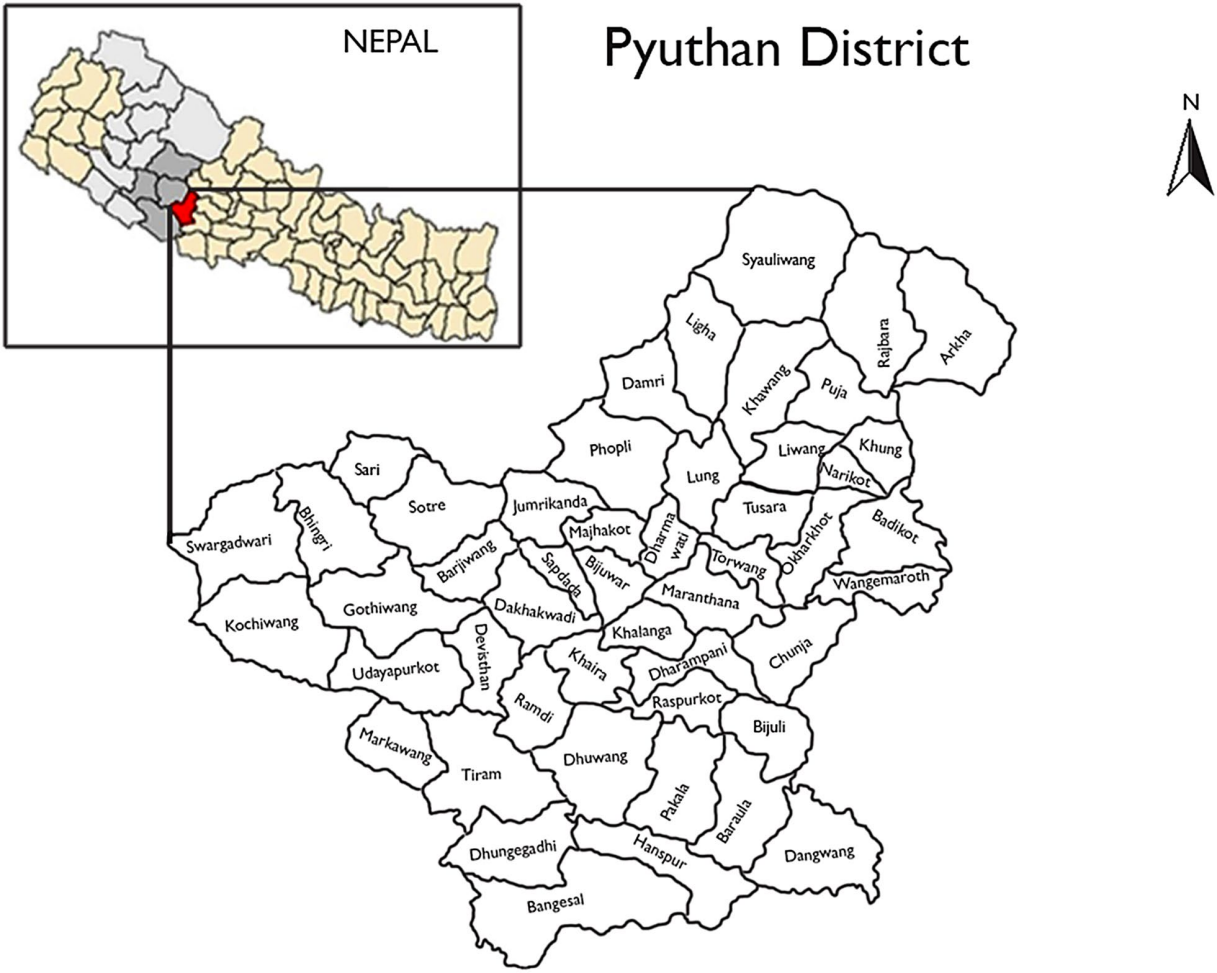
Map of study district

As part of the inception phase of the mhBeF project, investigators undertook a formative study with policy level stakeholders at the national level in Kathmandu and other district level stakeholders in Pyuthan district, located in the mid hills of the Rapti zone in the mid-western development region of Nepal [37] (Fig. 1). The objective of this formative research was to examine domains considered central to the successful development and implementation of a comprehensive community-based mental health services package with a mobile health component.

Nepal is a developing country with a low income, ranking at 145 of 187 countries on the Human Development Index (HDI) in 2015 [38]. Nepal recently endured the People’s War fought between the Communist Party of Nepal (Maoist) and government security forces from 1996 through 2006 causing widespread psychosocial and mental health implications. In rural Nepal, moderate to severe depression symptom prevalence is reported at 33% and anxiety prevalence at 27% [9]. Pyuthan district was among six districts seriously affected by the Maoist insurgency when the first ceasefire was declared on July 21, 2001 and till date there are no mental health and psychosocial interventions in the district.

Nepal has made significant progress in formulating a Mental Health Policy (1996) and Mental Health Act (2006). However, implementation has been inadequate and needs to be strengthened. The availability of human resources for health is also very scarce; 0.18, 0.04 and 0.25 psychiatrists, psychologists and nurses per 100,000 population in the country [27]. The District health system in Nepal is based on primary health care, which is more or less a self-contained segment of the national health system. District health system provides preventive, promotive and curative services to the people living in the districts through the District Public Health Office, District Hospitals, Primary Health Care Centers (PHCC), Health posts (HP), and Sub Health Posts (SHP) at all levels of the healthcare system. Pyuthan district has 1-district hospital, 2-primary health care centers, 11-health posts, and 35-sub health posts, about 40-pharmacies and 3-Ayurvedic health facilities. The peripheral health care system health work force, mainly consist of mid-level health worker groups who are largely paramedical practitioners Health Assistants (HA), Auxiliary Health Workers (AHW) etc. and nursing and nursing associate professional’s group. This includes Staff Nurse (SN) and Auxiliary Nurse Midwives (ANMs). Paramedical practitioners are also known as prescribers, as they run the Out -Patient Department (OPD) in health facilities where Nursing and nursing associates are known as non-prescribers since they are only involved in patient care and midwifery. Along with these barriers intervention studies are lacking for people with serious mental disease in primary care.

### Sampling procedure

This qualitative study was conducted by combining semi structured Key Informant Interviews (KIIs) and Focus Group Discussions (FGDs) to assess the feasibility of developing and implementing an evidence-based comprehensive community-based mental health services (CCMHS) package for persons with severe mental disorders and epilepsy (PWSMDE). This mixed method was applied for the purpose of obtaining complete data and comprehensive understanding of the study findings. The research question focused on what opinions and perceptions on CCMHS, regarding specific components in integrating mental health services into primary care with diversity of respondent’s opinions on different research theme selected from four categories of stakeholders; those working at the policy level, workers at health facility level, community level members and service users as shown in Table 1. The core interview guidelines were developed by “mhBeF” consortium and necessary adaptation was made for Nepal. Translation of the instruments was conducted following a systematic procedure developed for use in transcultural research [39]. A 7 day extensive training on research methodology including qualitative data collection methods (KIIs and FGDs) was provided to the field researchers prior to data collection. A greater proportion of the training was devoted to role playing and pre-test to ensure all field researchers were competent enough to collect good quality data. Necessary adaptations on interview guidelines were made after pre-testing. Purposive sampling was used for selecting the respondents for the FGDs considering on similar background/roles and hierarchy. This was in order to make discussions more comfortable for people. A total of 69 respondents participated in 9 FGDs with group sizes ranging between 6 and 12 respondents where homogeneity was observed in terms of occupation and location. Likewise, snowball sampling method was used to identify relevant stakeholders for the KIIs. To seek out the person with the other view 26 KIIs were conducted in total. Respondents were recruited through home visits and workplace visits (in the cases of policy level and health facility respondents) until no new additional information was generated from the interviews. All data was audio recorded along with note taking by extensively trained research assistants. They were closely supervised through daily reviews to discuss field experiences.

**Table 1:** Sampling by respondent type and research theme

Respondent type	Number of KII	Number of FGDs (n = number of participants)	Research theme						
			Healthcare workforce	Medicine supply chain	Patient support groups	Stigma	Family role	CoRPs role	Care and referral pathways
Policy level	(4)	0							
Director of PHC revitalization division	1	–	√	√					√
Mental health focal person in MOHP	1	–	√	√	√	√	√	√	√
District health officer	1	–	√	√	√	√	√	√	√
District hospital/Medical officer	1	–	√	√	√	√	√	√	√
Health facility level	(6)	3 (n = 23)							
Primary care workers (health facility incharge)	2	2 (n = 13)	√	√	√	√	√	√	√
Certified midwives/maternal health workers (ANM, staff nurse)	3	1 (n = 10)	√	√	√	√	√	√	√
Pharmacists	1	–	√	√		√			√
Community level	(8)	5 (n = 40)							
Community leaders*/VDC secretary/NGO representative	3	2* (n = 16)			√	√	√	√	√
Police	1	–			√	√	√	√	√
Traditional healers and herbalists	2	–			√	√	√	√	√
Teachers (secondary level or higher)	1	1 (n = 6)			√	√	√	√	√
Lay community health workers (FCHVs)	1	2 (n = 18)	√		√	√	√	√	√
Service users (SU)	(8)	1 (n = 6)							
Current or potential services users (PWSMDE)	4	1 (n = 6)		√	√	√	√	√	√
Family members and caregivers	4	–		√	√	√	√	√	√
Total	26	9 (n = 69)							

### Data management and analysis

Qualitative data obtained from KIIs to FGDs were transcribed in Nepali by field researchers immediately after each KII and FGD was completed. They were then translated into English by professional translators with over 2 years of experience in mental health. Translations were rigorously edited and crosschecked by two research officers who are fluent in Nepali and English before coding and analysis. This applied research findings aim to influence the Government of Nepal’s plans and policies through recommendations. There is an increasing trend of using framework analysis methodology in contrast to grounded theory which is developed to be is used in the context of applied policy research. Framework analysis (FA) allows for data collection, management and interpretation in a sequential fashion. FA applies a three-pronged approach to data; examining the data by themes, by type of respondent and by explanatory models available. The research team sought to understand what questions needed to be answered to inform how a project should deliver comprehensive mental health services within an existing health care system. What barriers would need to be addressed and who would be important drivers of successful integration of mental health services in routine primary care? Data was collected from important actors in the policy and community levels. Themes that emanated from discussions and factors explained the context for service delivery. The data was analyzed thematically using the framework analysis method [40]. It was cleaned, merged with field notes to make final transcripts and coded. A preliminary coding framework was developed based on prior themes and emerging themes. Code filter was developed putting the highlighted data in the categories from the interview data. A preliminary coding framework was pilot tested on 25% randomly selected data and analyzed by two different experienced researchers. It was then adapted, changes were made when necessary and the final coding framework was determined. This final coding framework was applied to all data sets. NVivo 10 software was used for coding and charting.

## Results/thematic findings

**Fig. 2:**
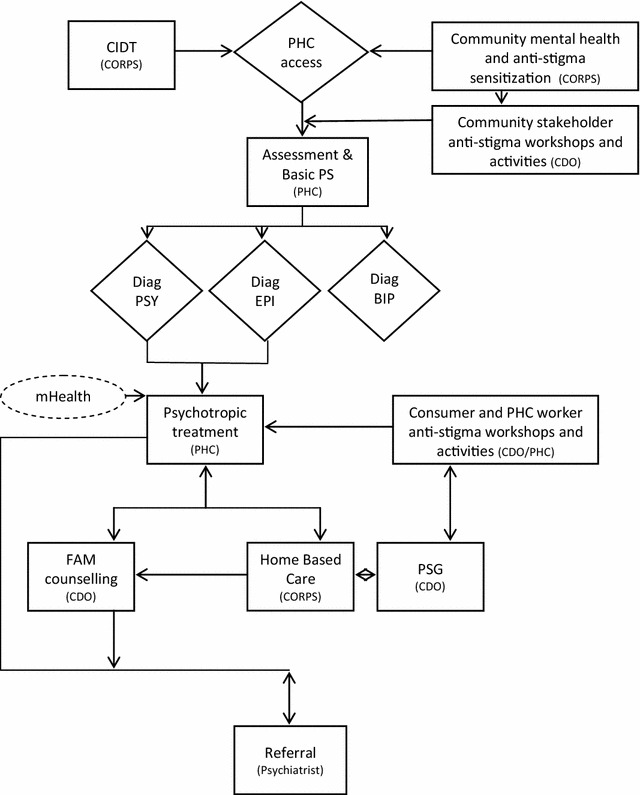
Flow chart: mhBeF intervention model

Major findings of the studies are presented in the different themes that emerged after data analysis. Table 2 summarizes the demographics of the final sample. Figure 2 summarizes flow chart: mhBeF intervention model

**Table 2:** Participant demographics

Socio-demographic characteristics	Key informant interviews(n)	Focus group discussions(n)	Total
Sex			
Male	18	28	46
Female	8	41	49
Age			
Up to 24	1	4	5
24–59	19	63	82
60 +	6	2	8
Age (mean)	–	–	47.68
Education			
Literate	4	10	14
Secondary	7	24	31
Intermediate (high school)	5	20	25
University	10	15	25
Religion			
Hindu	26	69	95
Occupation			
Health workers	8	23	31
Teacher	1	6	7
Agriculture	8	22	30
Female community health workers	2	18	20
Government officials	4	–	4
Others (NGO worker, political leader, traditional healer, etc.)	3	–	3
Total	26	69	95

Table 3 summarizes the major study findings and recommendations. The italicize text that follows are supported by actual statements that were given by the participants.

**Table 3:** Summary of study findings and recommendations

Level	Barriers identified for mental health service integration in routine primary health care	Study recommendations for adaptation of mhBeF model
Policy level	Inaccessible Mental Health Services in the community	Revision on Government National Treatment Guidelines and protocol
	Limited authority for Prescription of psychotropic drugs	Task shifting to PHC health workers
Health facility level	Knowledge and skills gap of Primary Health Care (PHC) health workers on mental health	Content for myths and facts related the mental health and treatment
		Duration of mental health training
		Use of mobile technology for effective mental health service delivery
Community level	Social stigma	Community resource persons involvement in mental health service delivery
	Lack of social support for people with mental health problems	Patient support group formation
		Family involvement in treatment and management
		Development of referral mechanism

### General mental health situation in Pyuthan district

Mental health services for example, general counseling and pharmacological treatment for mild depression, anxiety and insomnia are available in the district hospital. However, inpatient hospitalization services for severe disorders were nowhere to be found throughout the district. Respondent’s shared, that at the PHC level health facility only general counseling and symptomatic treatment of minor illness and referral services are provided. Most of the respondents reported that only severe psychotic problems are considered as mental health problems in the community. Although not common, mental health cases are often witnessed by the respondents and had different explanations for its causes and symptoms. There is a general belief that stress and tension, weak physical health/illness, torture, social change, improper ritual practices (*labeled locally as “deuta risako”),* poverty, unemployment, lack of education, economic problems and violence are considered as causes that can put a person at high risk of developing mental health problems which, can also lead to suicide. They generally have the view that treatment cannot fully cure mental disorders, since mental disorders are seen as the result of committing sinful activities, witchcraft or a curse. Thus, the community generally seeks treatment from traditional healers rather than from medical personnel.

*“People believe more to traditional healer because people say “boju lageko” to the person having mental problem. So they go to traditional healers at first then only come for medication in health facilities and if they do not recover from here, they go outside of district”.*

FGD-Health Facilities Managers

Due to stigma against mental health problems, most of the people suppress their problems in their initial stage which in turn causes difficulties in identification and early intervention for proper management. However, when the problem becomes more severe, the symptoms are overt and easily identifiable. Community people often use terms that carry a derogatory connotation when referring to people suffering from mental illness. They include terms like *mad, crazy, psycho, pagal, baulaha, taar khuskeko, jhalla, dimag navayeko, baudhik apangata, bojhu lageko*, *Aaushi purnima lageko, Khuskhet* and sometimes the terms used vary per severity of the problem. The study also revealed that derogatory terms were directed more towards marginalized groups (women, dalits, poor, ethnic minorities) adding yet another burden of stigma on people who are already at a disadvantage.

*“In our community people use terms like; mad, jhalla, pagal, khuskhet and it is usual in our community to discriminate against those people having mental problem who have low economic status, whereas, people from high economic status do not generally face stigma.*”

KII-Traditional Healer

### Challenges/barriers to integrate mental health services in routine primary health care of the district

Mental health services are not given high priority in Nepal by government. There is no provision of mental health and psychosocial services in the primary health care setting. Although, evidence on the efficacy and cost-effectiveness of specific interventions for mental disorders by primary health care professionals is now available, information on how these specific interventions can be combined into integrated packages in routine primary health care is lacking. Therefore, participants were asked on barriers and challenges to integrate mental health care plans in their district setting.

#### Inaccessible mental health services in the community

Mental health services were not available in the community setting where it was reported as one of the major barriers to integration by all the respondents. Due to unavailability of treatment services people travel to major cities and even India. Some of the respondents also mentioned geographical constraints as a major challenge for seeking services in the community, reaffirmed by the quotes below.

*“There is no treatment facility in the community. People need to go out of district. So, there are barriers like poverty and inaccessibility.”*

KII-Police Inspector

Although some antipsychotic drugs are listed under the country’s essential drug list, the government does not have provision for distributing them under Primary level health facilities.

*“Medicines for minor problems like headache are available but medicines for mental problems are not available in the health facili*ties”

KII-Auxiliary Nurse Midwife

It was found that community people do not seek treatment for general mental illness unless the problem is severe. They believe that treatment cannot fully cure mental disorders and that it is not worthwhile. They tend to prioritize the treatment of physical illness over mental problems.

*“Even though the family member knows about their problem, they do not provide them treatment soon enough. There is a trend of locking them in a room or chaining them. Families do not think of providing them treatment but when they do, they prefer to take them to traditional healers.”*

FGD-Auxiliary Nurse Midwife/Maternal and Child Health Worker

Community people still believe that mental problems are due to evil spirits and cannot be cured through hospital-based treatment. Given this misconception and belief in superstitions they usually go to traditional healers for treatment where they are told to appease the Gods by sacrificing animals such as goats and chickens. A case in point has been aptly put out in an FGD with health facilities in charge.

*“Our community does not believe that a mental problem is also a health related problem. Rather, they believe it is the result of an evil spirits and seek help from traditional healers. They clean themselves (chokho parne) and worship to ward off the evil spirits. Only when the problem gets severe do they come to us seeking help.”*

FGD-Health Facilities Manager

According to participants’, myths and misconceptions regarding mental health prevails in the community due to lack of awareness about the ability to treat mental illness. This misbelief is which seems one of the major barriers for seeking treatment for mental illness in the community and integrating mental health services in the district.

#### Knowledge and skills gap of primary health care health workers on mental health

It was admitted by PHC health workers that they do not possess adequate knowledge and skills in mental health since it is a very different field. Majority of respondents gave more emphasis on content development of the training package to ensure that all health workers are able to describe basic mental health concepts, deal with mental patients and have knowledge about mental health drugs.

However, they also stated that if PHC health workers were provided adequate training they would be able to provide mental health services such as counseling, detection, diagnosis, medication and stepwise management of mental disorders.

*“Since this is a different field, health workers may possess low knowledge. We work in all areas and there is no specific area for mental health. It would be better if we received trainings on identifying people suffering from mental problems and identifying the stages of the mental problems, as there are many people in our VDC who are in different stages of mental disorder. We also want training on how to manage problems in different stages to prevent them from going to another stage.”*

KII-Auxiliary Health Worker

#### Limited authority for prescription of psychotropic drugs

Mental health services have yet to be integrated in PHC level health facilities. Nepal’s national treatment protocol and drugs policy does not provide PHC health workers the authority to prescribe antipsychotic drugs. This was one of the major concerns laid by the policy level to PHC level health workers participants for mental health integration in routine health services provision.

*“Psychotropic drugs are on the essential drugs list but health workers like AHW have limited authority to prescribe these drugs. For example, doctors can only prescribe antibiotic drugs. Health workers may be able to diagnose the problem but they may not have the authority to prescribe the drug*.*”*

KII-Ministry of Health and Population: Section Officer

#### Social stigma

People suffering from mental illness are viewed as highly pathologized people, where people either scorn them or display callous attitudes towards them. Mental illness carries social stigma and therefore people do not want their problem to become known within the community. Because of self-consciousness they might stop visiting service centers.

*“Some people don’t visit health facility again because they are afraid that community people will know about their problem and discriminate against them. They also don’t want to return to health facility because they think they are more safe and better cured if they seek care from big hospitals.”*

KII-Maternal and Child Health Worker

As it was revealed from the study, community people are reluctant to associate with people suffering from mental health problems. They are not considered eligible to take part in social activities and festivities, and are a neglected category of people in the community.

*“People with mental disorders are mistreated mostly in social activities like, meetings, social gatherings, festivals and ceremonies. People either tease or scold if a person with a mental disorder participates in such social activities.”*

KII-NGOs Representative

According to the participants, this widely spread stigma in the community might be one of the challenges for integrating mental health services in the primary health care system.

#### Lack of social support for people with MH problems

People with Mental Disorders (PWMDs) are a part of the community but because they may not be able to contribute economically, or socially, they are often viewed as a burden in the family and society. Instead of supporting the PWMDs, respondents sometimes felt satisfaction in seeing their condition since they did not share a cordial relationship with the person or their family members.

*“When we were healthy we gave support to the society and now we are ill. So, I feel that our society is also ill because there is no one giving us proper suggestions for our condition.”*

FGD-Service Users

These circumstances show integration cannot be successful unless there is community support for people with mental health problems. This should be taken in consideration in the design of the intervention for the community based psychosocial and mental health disorders services.

### Receptivity to components of mhBeF model for improving access to mental health services

In addition to exploring barriers and challenges as described above, participants were also asked about the specific proposed components of the mhBeF intervention model. These key components included (1) task shifting mental health services to primary care and community health workers; (2) use of mobile technology for mental health service delivery; (3) establishment of patient support groups; (4) development of a new cadre of mental health workers: CoRPs; (5) family involvement in care; and (6) establishment of community-based referral mechanisms. Figure 2 provides the mhBeF conceptual model.

#### Task shifting for PHC health workers

Health workers at every level need basic health training on mental health issues. Type and content of training vary upon the health worker’s role within the health system and the services they provide. Most respondents gave more emphasis on content development of the training package in order to ensure that all health workers are able to describe basic mental health concepts, deal with mental patients and have knowledge about mental health drugs.

Almost all PHC level health workers answered that task shifting was acceptable and practicable but as they were not familiar with mental health disorders and its management aspects, they needed intensive training in the subject matter from mental health specialists. Most of respondents said that mental health services can be provided alongside the primary care that they currently provide.

*“Health facilities are able to provide mental health service but PHC staff does not have adequate knowledge about mental health because their syllabus does not adequately cover context on mental health. Patients, from those having general anxiety to those having severe mental disorders, visit PHC. Thus, PHC staffs should be provided trainings to enhance their ability to identify such disorders.”*

KII-Auxiliary Health Worker

*“It would be acceptable. We do not have any knowledge on mental health. So, I feel if I had knowledge on that sector then I would have provided treatment to them. I have to call other people to discuss the problem of the patient. So, I feel if I had known these things earlier I would have been able to provide them treatment. That’s why I think it would be acceptable for PHC worker*.*”*

KII-Staff Nurse

Although it is an additional task, health workers think that it falls under their responsibility to provide such services. Some of the respondents mentioned that health workers have their own task and that additional tasks can be burdensome. Therefore, they should be designed in manner that can be handled by health workers.

*“It has two sides. If this task is added by government then they will accept the task, thinking this is their job, but if NGOs add tasks to their job then they need incentive to do it. They think NGOs are consuming their time. They may do it initially but, if they are not provided facilities, they may stop doing it.”*

KII-Sr. Auxiliary Health Worker

Overall, respondents involved in the health system are cautious about task-shifting. Respondents at the HF level are often concerned about PHC workers’ capacity to go beyond counseling or referral roles. An AHW and MOPH respondent both agree with this position and said that PHC workers should not prescribe treatment (i.e. medication). This situation does not end here as personnel associated with the MoHP respondent repeated that they wanted medical doctors to diagnose the case.

*“Now in this situation we should focus on capacity building of health workers. Regular training and orientation should be given to them. We have been planning to provide the diagnosis facility at the level where medical doctors are available. And lower level health facilities like health post should do referral, counseling, follow*-*up and treatment.”*

KII-PHC Revitalization Division, Director

#### Use of mobile technology for effective mental health service delivery

This theme is centered around use of mobile phones for providing health care services and people’s perceptions, along with if they are familiar with digital health endeavors. Mobile technology was mostly taken positively even though it is a fairly new concept in the healthcare field. Even then, there were some responses that showed negative opinions about mobile technology. Concerns were mainly centered around the associated expenses and technical problems. Nevertheless, most health workers were interested and willing to try new forms of technology to aid their work as they were impressed with the idea and thought that this concept was a very practical and effective one. But among them, some raised concerns as to whether the service provider would be able to provide this service.

*“It will be feasible if people are able to use it. Documented cases can be easily searched and viewed. It will make the work of diagnosis much easier. If the people are able to use it then it will turn out to be a good idea.”*

KII-Staff Nurse

*“First thing that comes here is the equipment, mobile set is needed. The network (tower) is not good in Nepal. Network is not reachable or is in a very poor condition. We cannot talk clearly in mobiles phones. For its effective implementation, budget is needed and these are the constraining factors.”*

KII-District Health Officer

A few participants mentioned the non-feasibility and non-effectiveness of the service. This was reported since they believed t the health worker lacked the capability to take on this new task.

*″I don’t think it is feasible. Not all health workers are capable enough to send text messages to their supervisor and familiar with the operating system. I don’t think it would be feasible and effective”.*

KII-Auxiliary Nurse Midwife

#### Patient support group formation

No PSG currently existed in the project district and thus, they felt a need to form such groups. Respondents thought that formation of groups of mentally ill persons and their families would give them a forum where they could share their problems and experiences, and, enable each other through learning and sharing. They believed that such groups help to develop a collective front in order to raise their voice and fight against stigma in the community, where a considerable number of people still do not seek care and medication due to stigma.

*“They can share their own experience about how they can resolve the problem, the symptoms they had and about their treatment and inspire other people with mental illness. They can also suggest to their family member/care giver, in a positive way, how to support the person with a mental problem.”*

KII-Political Leader

Some respondents also shared different opinions about sustaining such groups. They thought that there needs to be a provision to provide people with mental illness with suitable skills to generate small-scale businesses and operate a saving credit group, so that they would not have to depend on external support.

*“The ill people should be given opportunity to learn different skills, for example, they may engage in poultry farming and kitchen gardening if they have received necessary training on that.”*

FGD-Health Facility Managers

A majority of the respondents were doubtful of running such groups due to widespread stigma toward mental illness. Besides stigma, they suggested that financial challenges need to be taken into consideration as an important barrier to running PSGs.

*“The group will experience obstacles in an early phase. Community people will not view such groups positively until the condition of people with mental problems has improved.”*

KII-NGO worker

#### CoRPS involvement in mental health service delivery

Participants thought that involving CoRPs in mental health service delivery may assist in reaching target groups. But before involving them, it was reported that these groups should be oriented about their role and importance in supporting people with mental health problems. The respondents stated that orientation and training should cover information about mental disorders, treatment services and other necessary support. The best way of involving CoRPs in the community would be through awareness programs and activities along with providing supportive roles in treatment and counseling.

*“They can disseminate information about mental health in the community. They can also provide support in identifying problems and offer appropriate referrals as well as counseling and psychosocial support.”*

KII-Teacher

#### Family involvement in treatment and management

Family plays a major role in delivering appropriate treatment to people with mental illness. Participants agreed that family is the most responsible for handling all the affairs related to people with mental health problems. Although families may never want to see their own member suffer, and despite their willingness to help, families with poor economic conditions are unable to seek proper care due to monetary constraints. Superstition and stigma demoralizes them and act as obstacles to care. Families have an important role in maintaining a co-operative living environment for people with mental problems. Therefore, given the pivotal role of families in mental health, there is a necessity to increase their awareness.

*“Family has a big role. Family should have supportive feelings for their ill member. Mental problems can be cured if there is good co*-*ordination between family members and their ill member. This is not an incurable disease. If the family is loving and caring for their member who is having problems then s/he might improve. Family’s role is to understand their desire, make them happy, fulfill their wants and make them satisfied. Love and care can increase their confidence level.”*

KII-Service User Care Giver

#### Development of referral mechanism

What also emerged from the study was that the community is not familiar with the treatment center and those seeking care are mostly referred to nearby health facilities. The general belief is that referring someone with mental disorders for medical treatment is akin to thrusting them towards stigma and discrimination from the community. As was revealed in the FGD with FCHVs, people feared that if the community finds out about their problems, they may face discrimination. Therefore, people secretly visited the FCHVs to get information regarding services. Apart from this, mental health services are not available in the district and people y are usually referred to Kathmandu, Rachi and Lucknow, where expert services are available. According to them, a referral center is necessary and should be established within the district so everyone can afford, and have easy access to those centers.

*“It would be better if there is place to refer the patient during the initial phase of symptoms that could prevent the problem from becoming severe. We need to emphasize preventive counseling. We need to establish treatment centers for severe mental health problems and advertise them through hoarding boards. In addition, awareness programs should be organized by NGOs like TPO Nepal in schools. This helps to identify people with mental disorders and to scale up treatment service.”*

KII-NGO Workers

## Discussion

The goal of this formative qualitative study was to identify resources, challenges, and potential barriers to implementation of CCMHS model, a community- and primary-care based mental health program. Key informant interviews and focus group discussions were utilized to identify both general resources and barriers and reactions to specific components of the CCMHS model. Key stakeholders include health systems personnel, health workers, potential beneficiaries of mental health services (consumers), and family members of persons living with severe mental illness. This study differs from prior formative studies [28, 29] because the current formative work focuses on a program for severe mental illness and includes novel components such as patient support groups (PSGs) and mobile electronic medical health documentation and decision aids (mHealth).

The formative research supported the expectation that there would be a lack of mental health services in primary health facilities even when community has recognized great need. Both community members and health workers reported the burden of untreated severe mental health problems. Of additional concern, both community members and health workers endorsed the belief that mental illness was not treatable. They think that treatment cannot fully cure mental disorders and it is worthless treating them. They tend to prioritize the treatment of physical illness over mental problems. Similar findings were documented in the previous studies [41–43].

Due to lack of awareness, some malpractice, such as chaining or locking in persons with mental illness still persists in the community [44]. The prevalence of stigma and discrimination regarding the mental health problem is widespread and community people do not seek treatment for general mental illness unless the problem is severe [28, 44 **]**. It was reported that most of the community people would opt to receive treatment from the traditional healers rather than medical treatment [28, 45–48] which could also be due to lack of availability and accessibility of medical services and costly medical treatment in the community [30].

People in poor economic conditions are not able to seek care regardless of their willingness [49, 50]. Superstitious belief and stigma comes in the way of seeking care [51–55]. Family has a vague role in maintaining co-operative environment for people with mental problem. Therefore, families are in huge need of awareness regarding mental disorder [28].

Almost all respondents answered that task shifting was acceptable and practical for non-specialist health workers [56]. However, there were some concerns at the Health Facility level about giving full responsibilities to PHC workers, as some respondents believed that they do not have capacity to prescribe treatment whilst others (including PHC workers themselves). They believed this was an acceptable proposal and that it would be possible with adequate training in the subject matter from the mental health specialist. Most of the respondents gave more emphasis on: (1) content development of the training package and making it familiar to PHC health workers to identify and treat people with mental disorders, (2) periodic supervision from the mental health specialist, and (3) knowledge about the psychotropic drugs prescription [57].

Although, PHC workers showed willingness, some respondents mentioned that their work schedule and division may be obstacles to perform these additional task [29, 58].

Formation of patient support groups of mentally ill persons and their families give them space to place share their problems, experiences, and strengthen each other through learning and sharing. Such groups also help to develop a collective strength of persons with mental illness to raise their voice to fight against stigma in community [59, 60]. Many studies have recommended that formation of self-help groups, along with the family support is the major component in the process of integrating mental health services for severe mental disorders in PHC levels and community settings [44, 61].

Mobile technology, though a new concept in the sector of medication [62, 63], was regarded positively. Majority of the thought this could be fruitful with adequate training in further equipping them in their tasks [64]. The concerns were mainly regarding the availability of internet network [64, 65], cost, over burden and low acceptability by health workers due to difficulties in learning this new task [66].

### Adaptations to CCMHS Package based on formative research findings

The formative research led to a range of recommendations for the adaptation and implementation of the CCMHS package:

1. *Duration of mental health training—*mhGAP is a 35 h curriculum. However, the low level of knowledge and limited training of primary health care workers (e.g., only 18–24 months of post-high school training) determined that a long curriculum with standard mhGAP was needed. Therefore, a 9-day primary care worker training was instituted. This was based on a 9-day training, which had been developed for primary care workers through PRIME in Southern Nepal.
2. *Content for myths and facts related the mental health and treatment—*The formative research identified an array of beliefs salient in this region of Nepal that would be important to include for myths and facts. Issues related to transmission of severe mental illness, the role of *karma* (past deeds in past lives) on causing mental illness, and supernatural possession by spirits (Nepali: *bhut, boksi*) are all common beliefs among community members and health care workers.
3. *Availability of psychotropic medication and government medication policies*—Lack of psychotropic medication is a major barrier to establishment of services for severe mental disorders. Treatment programs for schizophrenia, bipolar disorder, and epilepsy that do not include medication raise serious ethical concerns about providing diagnosis without available treatment. Therefore, coordination with the government to assure medication availability is crucial. Prescribing practices represent another challenge, as even if medications are made available, the government limits what medications can be prescribed by HAs who are the highest level providers in most facilities in this region. Therefore, government permission for psychiatric prescriptions is needed after adequate training and in the context of ongoing supervision. Finally, the types of psychiatric medications are also relevant. The essential medicines list is limited to first generation anti-psychotic and anti-depressant medication. Therefore, explorations with the government regarding providing more recent medications with lower side effects are needed.
4. *Selection of CoRPs—*In the mhBeF model, a small group of CoRPs were to be selected from existing community health volunteers, or Female Community Health Volunteers (FCHV). However, the formative research revealed that (1) there was a widespread need for mental health awareness among all FCHVs and (2) there was no clear selection process in place within the government system for determining which FCHVs would take on additional responsibilities. Therefore, we decided to conduct a district-wide 2-day orientation for the majority of FCHVs to maximize general mental health awareness. Among the FCHVs participating in the 2-day orientation, we would select the top scorers on a post-test knowledge and attitudes evaluation.
5. *Technological challenges—*The formative research revealed limited familiarity among health workers regarding basic computing and mobile technology. In addition, there was a widespread electricity shortage and limited wireless network availability. Therefore, it would not have been adequate to only record patient data on the mobile devices. We decided to include a paper-based documentation system which had previously been piloted in Nepal in a different primary-care based mental health program [67].

## Limitations

This study was conducted in a district setting and diversity was observed in terms of socio-economy, culture, ethnicity and geography across the district and country. Since adopting the qualitative design, purposive sampling method was applied where there is less chance of interviewing a representative sample from the population and the findings may not be generalized to all of Nepal.

In addition, the interviews were carried out in Nepali language and transcribed and translated to English. In this process, some rich and important information may have lost their original meaning.

## Conclusions

This study explored the existing situation of the mental health services and various barriers in developing and implementing mental health care package s in Nepal’s the primary health care setting. The stakeholders shared their ideas and experiences with mental illness and its treatment, myths and misconceptions, stigma and discrimination, also showed eagerness for the introduction of a CCMHS package provisions of (1) Task shifting to PHC health workers by taking consideration of prescriptions and making available of psychotropic medicines in peripheral health facilities after revision of the national treatment protocol and guidelines. (2) Upgrading the existing knowledge and skills of the PHC health workers through conducting and adopting mhGAP mental health training’s contents (stigma, myths and facts), durations and using mobile technology for effective service delivery. (3) Integration of social support components such as PSG, family support in treatment, use of community resource person (CoRPs) in mental health service delivery assisting in reaching needy target groups and development of referral mechanism into the existing health care system to narrow the treatment gap in the sector of mental health and psychosocial in Nepal.

## Abbreviations

AHW: auxiliary health workers
ANMs: auxiliary nurse midwives
BIP: bipolar disorder
CCMHS: comprehensive community based mental health services package
CIDT: community informant detection tool
CDO: Community Development Officer
CoRPs: community resource persons
DHO: district health officer
EPI: epilepsy
FCHVs: female community health volunteers
FGD: focused group discussion
KII: key-informant interview
HA: health assistants
HP: health posts
LMIC: low and middle income countries
MCHW: maternal and child health worker
mhBeF: mental health beyond facilities project
mhGAP: mental health gap action program
MoHP: Ministry of Health and Population
NHSSP: National Health Sector Support Program
NGO: non-government organization
OPD: out-patient department
PHC: primary Health Care
PHCC: primary health care centers
PSY: psychosis
PWSMD: persons living with severe mental disorders
SHP: sub health posts
SN: Staff Nurse
VDC: village development committee
